# Systemic immune-inflammation index predicts post-thrombectomy outcomes and reveals a mediating role in the association between neurocardiac stress and prognosis: a multicenter study

**DOI:** 10.3389/fneur.2026.1824909

**Published:** 2026-05-29

**Authors:** Long Yang, Han Zhao, Qingtao Xie, Qitong Chen, Yehong Liu, Hailiang Li, Jun Lu, Bo Du, Jiheng Hao, Jiyou Yu, Chao Sun, Yu Liu, Yujia Zhai, Haibing Liao, Yiming Yin, Hui Chen, Qiuchen Zhao, Yu Feng

**Affiliations:** 1 Department of Neurology, Affiliated Hospital of Xuzhou Medical University, Xuzhou, Jiangsu, China; 2 Klinik Fuer Nieren-und Hochdruckkrankheiten, Medizinische Hochschule Hannover, Hannover, Germany; 3 Xuzhou Medical University, Xuzhou, Jiangsu, China; 4 Central Military Commission Joint Logistics Support Force 904th Hospital, Wuxi, Jiangsu, China; 5 Department of Neurosurgery, Liaocheng People's Hospital, Liaocheng, Shandong, China; 6 Department of Neurology, Fengxian People's Hospital, Xuzhou, Jiangsu, China; 7 Suining County People's Hospital, Xuzhou, Jiangsu, China; 8 Xuzhou New Health Geriatric Hospital, Xuzhou, Jiangsu, China; 9 The Second Affiliated Hospital of Xuzhou Medical University, Xuzhou, Jiangsu, China; 10 Medical University of Tianjin, Tianjin, China; 11 Department of Neurology, Xuzhou Central Hospital, Xuzhou, Jiangsu, China; 12 Department of Neurology, MassGeneral Institute for Neurodegenerative Disease, Massachusetts General Hospital, Harvard Medical School, Charlestown, MA, United States

**Keywords:** atrial fibrillation, inflammation, ischemic stroke, neurocardiac syndrome, nomogram, prognosis, thrombectomy

## Abstract

**Background:**

Systemic inflammation and neurocardiac stress are key determinants of poor outcomes after endovascular thrombectomy (EVT), yet their interplay remains poorly quantified. Existing models often rely on static factors and overlook the dynamic interaction between these two pathways. This study investigates whether the Systemic Immune-Inflammation Index (SII) statistically accounts for a portion of the association between atrial fibrillation with rapid ventricular response (AF-RVR)—a clinical marker of neurocardiac stress—and poor outcome in stroke patients undergoing EVT.

**Methods:**

In this multicenter retrospective study, 678 EVT patients were screened, yielding a derivation cohort of 515. Using LASSO and multivariable logistic regression, we developed a model incorporating AF-RVR, SII, and key clinical/procedural variables to predict poor 3-month functional outcome (mRS 3–6). Mediation analysis was employed to quantify the indirect effect of AF-RVR on outcome via SII. External validation was performed in 181 independent patients. The model was implemented as an online risk calculator.

**Results:**

Poor outcome occurred in 56.1% (289/515) of the derivation cohort. AF-RVR correlated with elevated SII. Patients with both AF-RVR and high SII had a significantly increased risk of poor outcome (adjusted OR 9.80; 95% CI, 3.46–31.43), with a graded risk across subgroups. Mediation analysis revealed that the indirect effect of AF-RVR through SII was significant (absolute risk difference 4.6 percentage points; 95% CI, 1.1–8.4). The model demonstrated good discrimination (AUC 0.789 derivation, 0.710 validation), calibration, and clinical utility on decision curve analysis.

**Conclusion:**

We developed and externally validated a nomogram integrating AF-RVR and SII to predict post-EVT outcomes. The prognostic association of AF-RVR was partially statistically accounted for by systemic inflammation. This tool supports early individualized risk stratification and underscores the importance of jointly assessing neurocardiac stress and systemic inflammation in post-thrombectomy care.

## Introduction

Endovascular thrombectomy (EVT) has transformed the treatment of acute ischemic stroke (AIS) due to large vessel occlusion, achieving high rates of successful recanalization (1). Nevertheless, nearly half of patients undergoing EVT fail to regain functional independence (1, 2), underscoring that neurological recovery depends on complex multisystem processes extending beyond vascular reperfusion (3).

Two interrelated systemic pathways have emerged as pivotal modulators of post-EVT outcomes: systemic inflammation and maladaptive neurocardiac responses, collectively described as stroke-heart syndrome (4–6). Systemic inflammation, quantifiable via indices such as the Systemic Immune-Inflammation Index (SII), aggravates secondary brain injury and impairs neuroplasticity (3, 7, 8). Accumulating evidence has established SII as a robust prognostic biomarker in ischemic stroke, with meta-analyses demonstrating that elevated SII is significantly associated with poor functional outcomes (3, 7). Concurrently, acute brain injury disrupts central autonomic control, triggering sympathetic overactivation that manifests as cardiac arrhythmias and myocardial injury (6, 9). This neurogenic stress may compromise cerebral perfusion despite successful recanalization, contributing to the “no-reflow” phenomenon (3, 10, 11).

Atrial fibrillation (AF) occupies a central role in these intertwined pathways. The presence of rapid ventricular response (RVR; heart rate >100 bpm) during AF in the hyperacute phase likely reflects intense maladaptive sympathetic activation—a “catecholamine storm” secondary to brain injury (4, 6, 12). Thus, AF with RVR (AF-RVR) may define a distinct high-risk phenotype, capturing both embolic risk and the severity of neuro-cardiac dysfunction. This is further supported by bidirectional crosstalk: sympathetic activation promotes pro-inflammatory cytokine release, while inflammatory mediators sensitize cardiac afferents, amplifying both arrhythmogenesis and systemic inflammation (13–15). Consequently, the coexistence of AF-RVR and elevated systemic inflammation may identify a patient subgroup with compounded vulnerability and worse recovery potential.

Existing prognostic models for EVT, including THRIVE and DRAGON, primarily incorporate baseline clinical and procedural variables but largely neglect dynamic systemic responses and the prognostic nuances of specific cardiac phenotypes (16, 17). To date, no widely used tool integrates detailed AF phenotyping (e.g., AF-only versus AF-RVR) with validated systemic inflammation markers to comprehensively predict post-EVT outcomes.

To address these gaps, we aimed to: (1) evaluate the differential prognostic impact of AF-RVR in a modern EVT cohort; (2) develop and externally validate a nomogram integrating neurovascular factors, systemic inflammation (SII), and neuro-cardiac status; and (3) explore whether systemic inflammation statistically accounts for part of the association between AF-RVR and poor functional outcomes, providing insight into the stroke-heart syndrome.

## Methods

### Study design and population

This multicenter, retrospective study aimed to develop a prediction model and adhered to the TRIPOD guidelines for predictive modeling (18). The study protocol was approved by the Institutional Review Board of the Affiliated Hospital of Xuzhou Medical University (approval number XYFY2023EVT001), which waived the requirement for informed consent due to the retrospective nature of the study.

We screened consecutive adult patients (>18 years) with acute ischemic stroke resulting from large vessel occlusion (anterior or posterior circulation) who underwent endovascular thrombectomy (EVT) within 24 h of symptom onset. These patients were recruited from seven tertiary stroke centers in China between January 2023 and October 2024. Key inclusion criteria were: (1) confirmation of occlusion via computed tomography angiography (CTA), magnetic resonance angiography (MRA), or digital subtraction angiography (DSA); (2) EVT performed within 24 h of symptom onset; and (3) availability of admission electrocardiogram (ECG) and complete blood count (CBC) data. Patients were excluded if they had pre-stroke disability (defined as a modified Rankin Scale score >2) or lacked essential laboratory or heart rate data.

Clinical, procedural, and laboratory data were retrospectively collected from electronic medical records using a standardized case report form. Additionally, a temporally independent external validation cohort, meeting identical eligibility criteria, was recruited from a separate center between December 2024 and June 2025.

### Data collection and definitions of key predictors

Demographic characteristics, vascular risk factors, stroke severity (assessed using the NIH Stroke Scale [NIHSS]), procedural timelines (onset-to-puncture time, procedure duration), revascularization outcomes (modified Thrombolysis in Cerebral Infarction [mTICI] score), and admission laboratory values were extracted by trained researchers who were blinded to patient outcomes. Data collection was performed using a standardized case report form.

### Definitions of key predictors

Atrial fibrillation (AF) was defined by one of the following criteria: a documented history of AF, evidence of AF on the admission standard 12-lead electrocardiogram (ECG), or AF identified during ≥24 h of continuous cardiac monitoring following admission. The admission heart rate was recorded as the initial ventricular rate confirmed on the ECG obtained at the time of hospital arrival, prior to any sedation, EVT procedure, or rate-control therapy. Patients were categorized into three groups: no AF, AF only (heart rate ≤100 bpm), and AF with rapid ventricular response (AF-RVR; heart rate >100 bpm), consistent with current clinical guidelines (19).

The Systemic Immune-Inflammation Index (SII) was calculated using the formula: (Platelet count × Neutrophil count) / Lymphocyte count, based on the first complete blood count obtained upon admission (20). For model development, SII was dichotomized at the median value of the derivation cohort (1012.23 × 10^9^/L), creating two groups: High SII and Low SII. This threshold approximated the risk cutoff identified in prior studies [e.g., 1010.5 × 10^9^/L in Qian et al. (7)], providing a practically interpretable stratification within our cohort. We acknowledge that the use of a data-driven threshold is a limitation, and sensitivity analyses using continuous SII or literature-derived cutoffs would be valuable in future studies.

### Outcome

The primary outcome was poor functional outcome at 3 months, defined as a modified Rankin Scale (mRS) score of 3–6 (versus functional independence, defined as mRS 0–2). Functional status was ascertained from outpatient records or structured telephone interviews.

### Statistical analysis

This study employed a convenience sampling of consecutive eligible patients. The derivation cohort included 515 patients, with 289 (56.1%) experiencing the primary outcome. Based on established guidelines recommending at least 10 outcome events per predictor, this sample size was adequate for developing a model with up to 8 predictors.

Continuous variables are presented as mean ± standard deviation (SD) or median (interquartile range [IQR]), and categorical variables as counts (percentages). Group differences were assessed using appropriate tests (*t*-test, Mann–Whitney U, chi-squared, or Fisher’s exact test) depending on data distribution and type.

Model development utilized the derivation cohort. Candidate predictors with *p* < 0.10 in univariable logistic regression were subjected to Least Absolute Shrinkage and Selection Operator (LASSO) regression with 10-fold cross-validation for variable selection. Predictors with non-zero coefficients were entered into a multivariable logistic regression model (“LASSO Model”). An Interaction Model incorporated binary atrial fibrillation with rapid ventricular response (AF-RVR) and its interaction with high SII to assess potential synergistic effects.

Model performance was evaluated by discrimination (area under the receiver operating characteristic curve [AUC]), calibration (calibration plots and Hosmer–Lemeshow test), and clinical utility (decision curve analysis [DCA]). Internal validation was conducted using 1,000 bootstrap resamples, while external validation applied the final model with fixed coefficients to an independent temporal cohort.

To explore whether systemic inflammation accounts for part of the association between AF-RVR and poor outcome, mediation analysis was performed using the R package mediation (version 4.5.1) in R 4.3.2. High SII (dichotomized at the median) served as the mediator, and AF-RVR as the exposure. Two logistic models were fitted: (1) mediator model: High SII ∼ AF-RVR + covariates; (2) outcome model: poor outcome ∼ AF-RVR + High SII + covariates.

Covariates included in both models matched those in the final prediction model: age, >1 thrombectomy attempts, onset-to-puncture time, procedure duration, diabetes, and pre-EVT NIHSS score. Complete-case analysis was applied.

The average causal mediation effect (ACME), average direct effect (ADE), total effect, and proportion mediated were estimated on the risk difference scale (absolute percentage points) using 1,000 nonparametric bootstrap iterations to obtain bias-corrected 95% confidence intervals (CIs). This approach quantifies how much of the effect of AF-RVR on poor outcome is mediated through high SII versus being direct.

A two-sided *p* < 0.05 was considered statistically significant.

### Implementation as an online calculator

To enhance clinical utility and facilitate point-of-care decision-making, the final prediction model was implemented as an interactive, web-based calculator. The calculator, built using the Shiny framework for R, allows clinicians to input individual patient data and receive real-time, quantified risk estimates for poor functional outcomes at 3 months.

## Results

### Study population and baseline characteristics

A total of 515 consecutive patients were included in the derivation cohort. The primary outcome of poor functional status at 3 months, defined as a modified Rankin Scale (mRS) score of 3–6, occurred in 289 patients (56.1%). As summarized in Table 1, compared to patients with favorable outcomes (mRS 0–2), those with poor outcomes were significantly older, had higher baseline stroke severity (as assessed by NIHSS), experienced longer procedural timelines (onset-to-puncture time and procedure duration), and exhibited a higher prevalence of diabetes, AF-RVR, and an elevated systemic immune-inflammation index (SII > 1012.23) (all *p* < 0.05).

**Table 1:** Baseline characteristics of the derivation cohort stratified by 3-month functional outcome.

Variable	Total (*N* = 515)	Poor outcome (mRS 3–6, *N* = 289)	Good outcome (mRS 0–2, *N* = 226)	*P* value
Demographics				
Age, years, median (IQR)	67.0 (58.5–75.0)	70.0 (60.0–77.0)	66.0 (56.0–73.0)	<0.001
Vascular risk factors, *n* (%)				
Hypertension	262 (50.9)	160 (55.4)	102 (45.1)	0.021
Diabetes mellitus	100 (19.4)	67 (23.2)	33 (14.6)	0.015
Stroke characteristics				
Admission NIHSS score, median (IQR)	20.0 (12.0–30.0)	22.0 (15.0–30.0)	16.0 (11.0–25.8)	<0.001
Procedural characteristics				
Onset-to-puncture time, min, median (IQR)	350 (250–466)	380 (280–480)	310 (230–420)	<0.001
Procedure duration, min, median (IQR)	95 (70–125)	100 (80–140)	90 (60–116)	<0.001
Thrombectomy attempts >1, n (%)	142 (27.6)	94 (32.5)	48 (21.2)	0.004
Final mTICI 2b–3, n (%)	394 (76.5)	207 (71.6)	187 (82.7)	0.002
Neurocardiac and inflammatory markers				
Admission heart rate, bpm, median (IQR)	79 (69–92)	85 (74–98)	74 (66–81)	<0.001
AF with RVR (HR >100 bpm), *n* (%)	67 (13.0)	49 (17.0)	18 (8.0)	0.003
High SII (>1012.23), *n* (%)	258 (50.1)	185 (64.0)	73 (32.3)	<0.001
Neutrophil-to-lymphocyte ratio, median (IQR)	5.63 (3.25–9.41)	6.67 (3.47–10.61)	4.87 (2.90–8.27)	<0.001

Data are presented as median (interquartile range [IQR]) for continuous variables and number (%) for categorical variables. p values were calculated using the Mann–Whitney U test or χ^2^ test, as appropriate. AF indicates atrial fibrillation; RVR, rapid ventricular response; SII, systemic immune–inflammation index; NIHSS, National Institutes of Health Stroke Scale; mTICI, modified Thrombolysis in Cerebral Infarction.

A comprehensive set of baseline characteristics for the derivation cohort is provided in Supplementary Table S1. For external validation, a temporally independent cohort of 181 patients was utilized. The detailed baseline characteristics of this cohort are presented in Supplementary Table S2. The derivation and validation cohorts were broadly comparable in terms of key demographic and clinical variables.

### Clinical profile of the AF-RVR phenotype

Baseline characteristics of patients with and without atrial fibrillation with rapid ventricular response (AF-RVR) are compared in Supplementary Table S3. Patients with AF-RVR were significantly older and less frequently male 52.2%vs.70.8%,P=0.002 . They also had a markedly higher prevalence of cardioembolic stroke etiology (76.1% vs. 19.9%, *p* < 0.001). Additionally, patients with AF-RVR presented with more severe baseline stroke symptoms, as indicated by a higher NIHSS score 23.8±8.9vs.20.3±9.6,P=0.004 , and exhibited a more pronounced systemic inflammatory response. This was reflected in a greater proportion of patients with high SII 64.2%vs.48.0%,P=0.013 and a higher neutrophil-to-lymphocyte ratio 9.3±6.6vs.7.4±8.5,P=0.005 .

This distinct clinical profile—characterized by concurrent neurocardiac stress and heightened systemic inflammation—was associated with a significantly higher rate of poor functional outcomes at 3 months 73.1%vs.53.6%,P=0.003 .

### Model development: predictor selection and multivariable analysis

Variables that were significantly associated with poor outcomes at *p* < 0.10 in univariable analyses (Supplementary Table S4) were retained as candidate predictors. To minimize overfitting and select a parsimonious set of predictors, we employed Least Absolute Shrinkage and Selection Operator (LASSO) regression with 10-fold cross-validation (Supplementary Figure S1).

LASSO regression identified eight predictors with non-zero coefficients, which were subsequently included in a multivariable logistic regression model (LASSO Model, Table 2). The final model identified the following independent predictors of poor 3-month functional outcome: older age (adjusted odds ratio [aOR] per year increase: 1.034, 95% confidence interval [CI] 1.016–1.052), higher pre-EVT NIHSS score (aOR per point: 1.038, 95% CI 1.015–1.062), longer onset-to-puncture time (aOR per minute: 1.002, 95% CI 1.001–1.003), longer procedure duration (aOR per minute: 1.005, 95% CI 1.001–1.010), higher admission heart rate (aOR per bpm: 1.031, 95% CI 1.017–1.044), and high systemic immune-inflammation index (SII) (aOR: 3.225, 95% CI 2.130–4.901).

**Table 2:** Multivariable logistic regression and interaction analysis for predictors of poor 3-month functional outcome.

Variable	LASSO Model aOR (95% CI)	Interaction Model aOR (95% CI)	Stratum-Specific ORs (95% CI)
Age (per 1-year increase)	1.034 (1.016–1.052)***	1.037 (1.019–1.056)***	
Thrombectomy attempts >1	1.321 (0.991–1.766)	1.294 (0.967–1.741)	
Onset-to-puncture time (per min)	1.002 (1.001–1.003)**	1.002 (1.001–1.003)**	
Procedure duration (per min)	1.005 (1.001–1.010)*	1.005 (1.000–1.010)*	
Diabetes mellitus	1.646 (0.978–2.786)	1.641 (0.970–2.791)	
Admission heart rate (per bpm)	1.031 (1.017–1.044)***	1.040 (1.023–1.057)***	
Pre-EVT NIHSS score	1.038 (1.015–1.062)**	1.038 (1.015–1.062)**	
High SII	3.225 (2.130–4.901)***	2.963 (1.916–4.603)***	
AF-RVR	—	0.302 (0.106–0.858)*	
AF-RVR × High SII	—	2.404 (0.621–9.309)	
Stratum analysis			
Low SII & No AF-RVR			Reference
Low SII & AF-RVR			7.121 (2.341–39.471)
High SII & No AF-RVR			3.307 (1.198–10.717)
High SII & AF-RVR			9.797 (3.464–31.425)

Adjusted odds ratios (aORs) were obtained from multivariable logistic regression models. Interaction term P value = 0.205. *p < 0.05, **p < 0.01, ***p < 0.001. AF-RVR indicates atrial fibrillation with rapid ventricular response; SII, systemic immune–inflammation index; EVT, endovascular thrombectomy; CI, confidence interval.

Diabetes mellitus (aOR: 1.646, 95% CI 0.978–2.786) and multiple thrombectomy attempts (aOR per attempt: 1.321, 95% CI 0.991–1.766) were retained in the final model despite not reaching statistical significance after mfultivariable adjustment, as both variables were selected by LASSO regression and contributed to the overall predictive performance of the model.

### Investigating the interplay between AF-RVR and high SII: risk gradient analysis

**Figure 1:**
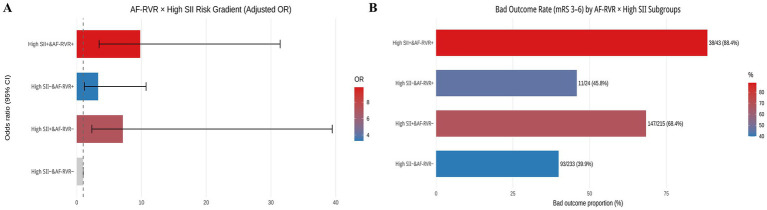
Risk gradient associated with combined AF-RVR and elevated SII for poor functional outcome after EVT. **(A)** Forest plot displaying adjusted odds ratios (aORs) with 95% confidence intervals for poor functional outcome across predefined patient strata. **(B)** Bar chart illustrating the observed proportion of patients with poor functional outcome (modified Rankin Scale [mRS] score 3–6) within each stratum.

To evaluate the potential interplay between neurocardiac stress and systemic inflammation, AF-RVR and its multiplicative interaction term with high SII were incorporated into the core LASSO-derived multivariable model. In stratified analyses adjusted for covariates included in the final model, a clear and graded increase in the risk of poor 3-month functional outcome was observed across strata (Table 2; Figure 1).

Using patients with low SII and no AF-RVR as the reference group, the adjusted odds of poor outcome were significantly higher among patients with high SII alone (adjusted odds ratio [aOR] 3.31, 95% CI 1.20–10.72) and were further increased among those with AF-RVR alone (aOR 7.12, 95% CI 2.34–39.47). The highest risk was observed in patients with concomitant AF-RVR and high SII (aOR 9.80, 95% CI 3.46–31.43).

This stepwise escalation in statistical risk was mirrored by the observed outcome proportions shown in Figure 1, with progressively higher rates of poor functional outcome across the reference, high SII alone, AF-RVR alone, and combined groups. Although the multiplicative interaction term did not reach statistical significance P=0.205 , the consistent risk gradient across strata suggests that the co-occurrence of AF-RVR and elevated systemic inflammation identifies a subgroup with particularly high vulnerability following thrombectomy.

### Mediation analysis: systemic inflammation partially mediates the AF-RVR–outcome association

As an exploratory analysis, we examined whether systemic inflammation may statistically account for part of the association between AF-RVR and poor functional outcome by conducting a mediation analysis adjusting for all covariates included in the final prediction model, with high SII specified as the mediator. These findings should be considered hypothesis-generating and interpreted with caution. (Supplementary Table S5).

On the absolute risk difference scale, the total effect of AF-RVR on poor 3-month functional outcome was 0.123 (95% CI, 0.002–0.258, *p* = 0.046), indicating that patients with AF-RVR had a 12.3 percentage-point higher absolute probability of poor outcome compared to those without AF-RVR. The average causal mediation effect (ACME) via high SII was 0.046 (95% CI, 0.011–0.084, *p* = 0.018), corresponding to an indirect effect of 4.6 percentage points. In contrast, the average direct effect (ADE) of AF-RVR—independent of high SII—was not statistically significant (0.077, 95% CI, −0.040 to 0.206, *p* = 0.210). Exploratory mediation analysis yielded a mediated proportion of 37.2% (95% CI, −14.8 to 241.0%, Supplementary Table S5); however, given the wide confidence interval crossing zero, this estimate is highly unstable and should not be interpreted as a precise or reliable quantitative summary. The average causal mediation effect (ACME) reached statistical significance, which may provide preliminary support for a potential mediating role of systemic inflammation, though these findings remain hypothesis-generating and require confirmation in future prospective studies.

**Figure 2:**
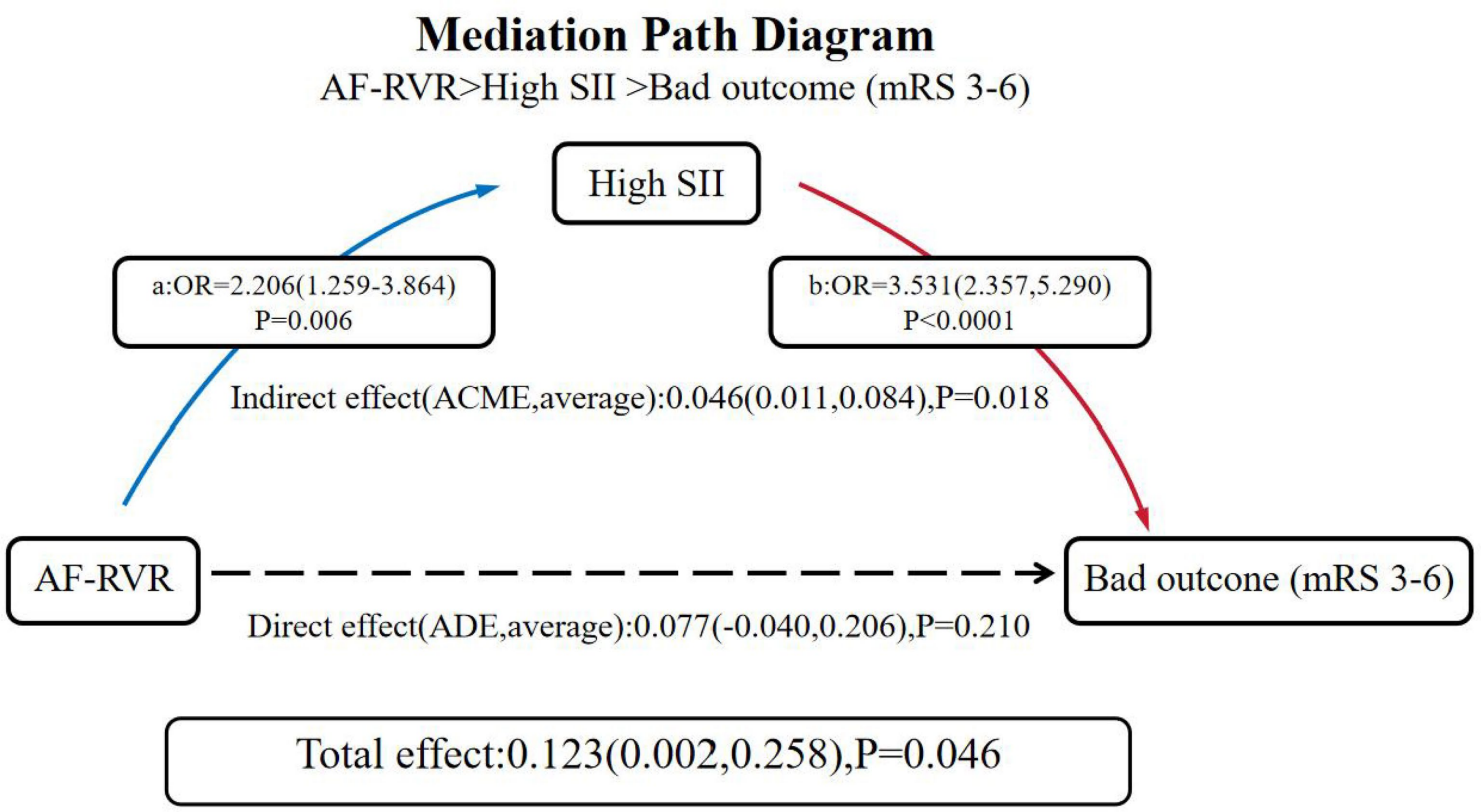
Causal mediation path diagram illustrating the role of high SII in the association between AF-RVR and poor 3-month functional outcome. Values on paths represent adjusted odds ratios (OR) derived from logistic regression models: Path a (AF-RVR → high SII): OR = 2.21 (95% CI, 1.26–3.86), *p* = 0.006; Path b (high SII → poor outcome, adjusted for AF-RVR and covariates): OR = 3.53 (95% CI, 2.36–5.29), *p* < 0.001; Path c’ (AF-RVR → poor outcome, adjusted for high SII and covariates): OR = 1.47 (95% CI, 0.78–2.76), *p* = 0.234. Mediation effects estimated on the absolute risk difference scale (bootstrap, 1,000 iterations): Total effect: 0.123 (95% CI, 0.002–0.258), *p* = 0.046; Indirect effect (ACME): 0.046 (95% CI, 0.011–0.084), *p* = 0.018; Proportion mediated: 37.2% (95% CI, −14.8 to 241.0%). All models were adjusted for age, thrombectomy attempts >1, onset-to-puncture time, procedure duration, diabetes, and pre-EVT NIHSS score. Abbreviations: AF-RVR, atrial fibrillation with rapid ventricular response; SII, systemic immune-inflammation index; ACME, average causal mediation effect.

Collectively, these results indicate that systemic inflammation statistically accounts for a portion of the association between AF-RVR and poor outcome. After adjustment for high SII and all covariates, the residual direct effect of AF-RVR was no longer statistically significant, suggesting that the arrhythmia phenotype itself contributes minimal additional risk beyond the inflammatory and neurocardiac disturbances it reflects. A graphical summary of this mediation pathway is shown in Figure 2.

### Model performance, validation, and clinical translation

**Figure 3:**
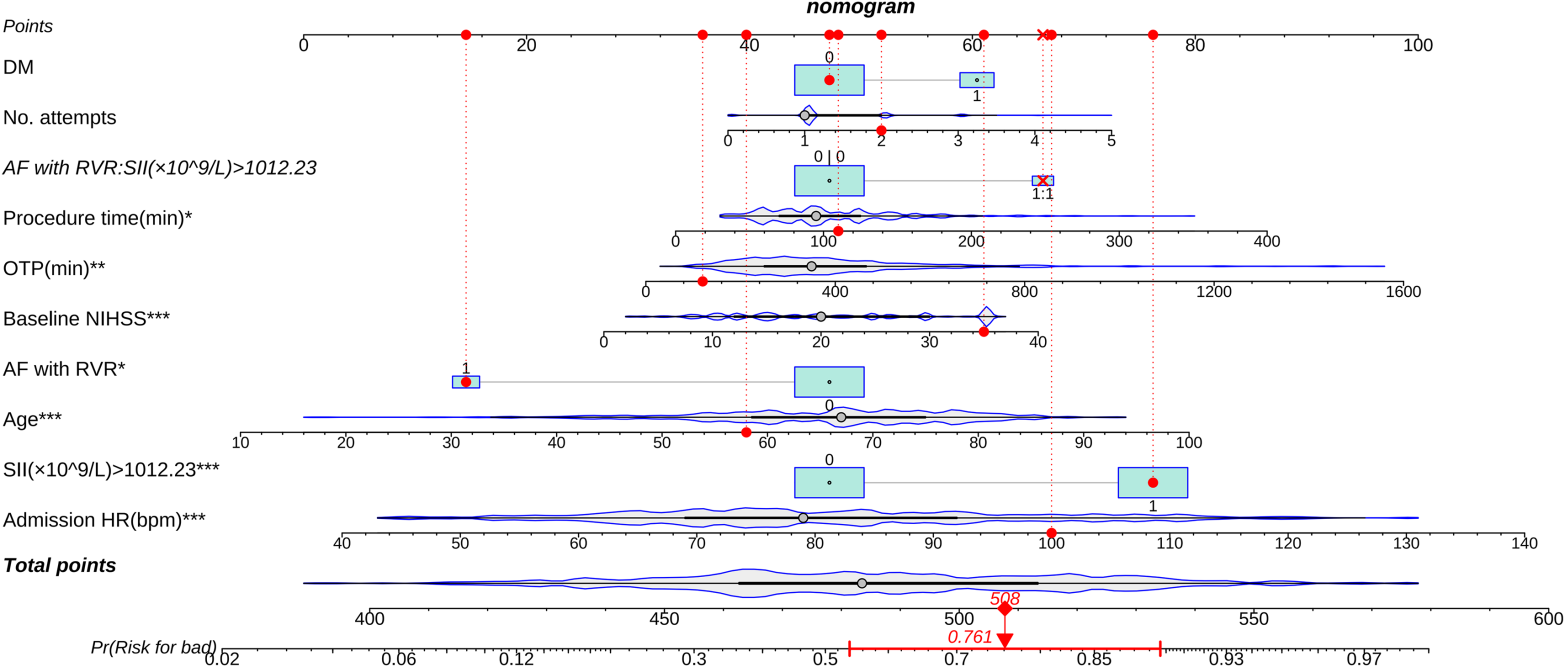
Nomogram for predicting poor 3-month functional outcome after EVT. The nomogram visualizes the final prediction model derived from the interaction analysis. To estimate an individual patient’s risk, locate each predictor value on its corresponding axis and draw a vertical line to the “Points” scale to obtain the assigned score. The points for all predictors are then summed to yield the “Total Points,” which correspond to the predicted probability of poor functional outcome on the lower risk axis.

**Figure 4:**
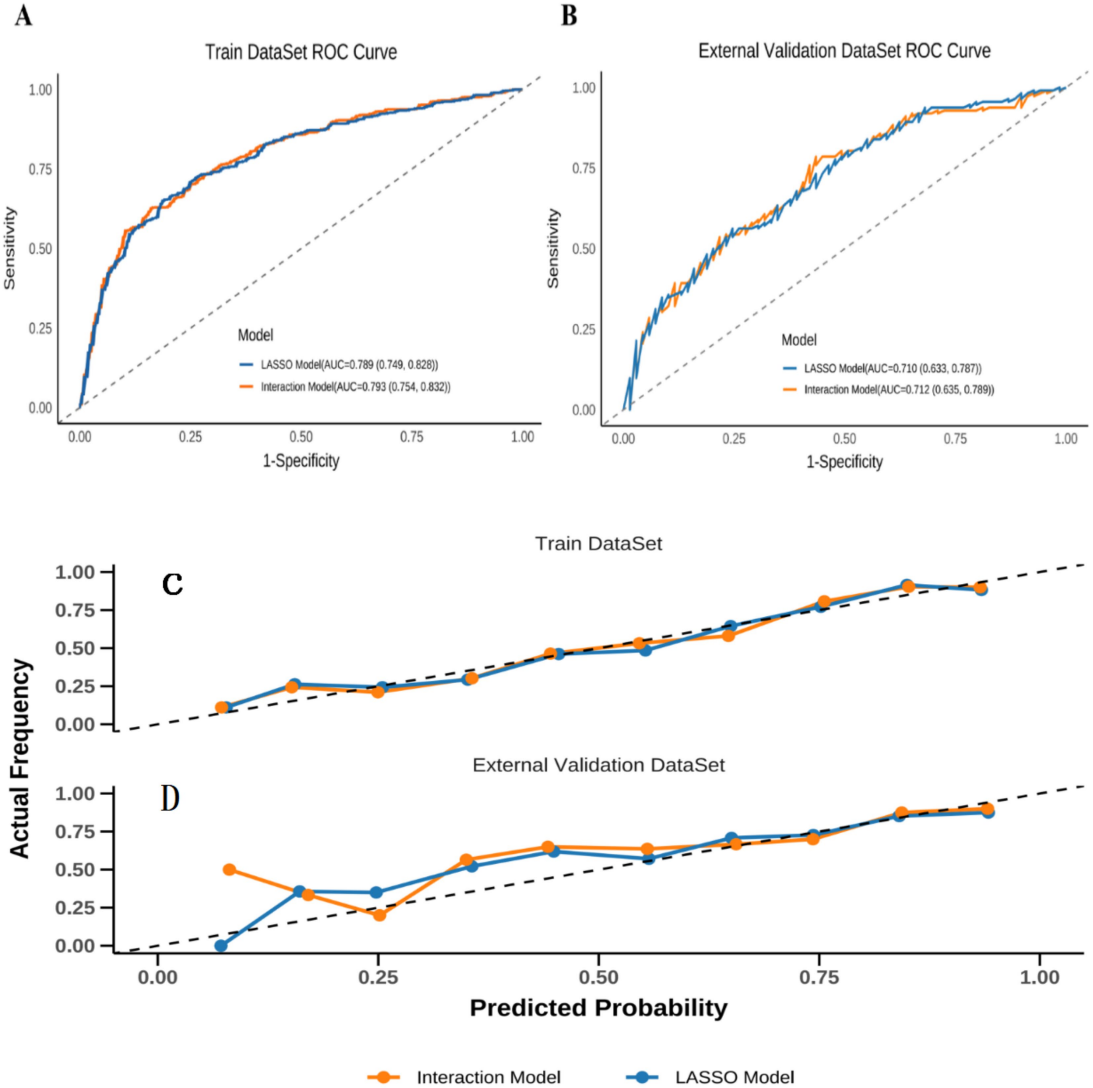
Discrimination and calibration of the prediction model in the derivation and external validation cohorts. **(A)** Receiver operating characteristic (ROC) curves for the LASSO model and the interaction model in the derivation cohort, with areas under the curve (AUCs) of 0.789 (95% CI, 0.749–0.828) and 0.793 (95% CI, 0.754–0.832), respectively. **(B)** ROC curves for the two models in the external validation cohort, with corresponding AUCs of 0.710 (95% CI, 0.633–0.787) and 0.712 (95% CI, 0.635–0.789). **(C,D)** Calibration plots for the interaction model in the derivation **(C)** and external validation **(D)** cohorts, demonstrating agreement between predicted probabilities and observed event rates of poor functional outcome. The Hosmer–Lemeshow goodness-of-fit test indicated adequate calibration (*p* = 0.870 and *p* = 0.078, respectively). The dashed diagonal line represents perfect calibration.

The final model was translated into a practical nomogram for individualized risk estimation (Figure 3). Its predictive performance was both robust and generalizable. In the derivation cohort, the model achieved an area under the receiver operating characteristic curve (AUC) of 0.793 (95% confidence interval [CI] 0.754–0.832), and in the external validation cohort, the AUC was 0.712 (95% CI 0.635–0.789) (Figures 4A,B; Table 3). Calibration plots demonstrated excellent agreement between predicted and observed outcomes in both cohorts (Figures 4C,D). Decision curve analysis further confirmed that the model provided a superior net clinical benefit across a broad range of decision thresholds when compared with “treat all” or “treat none” strategies (Supplementary Figure S2). To facilitate immediate clinical implementation, we developed an interactive, web-based risk calculator https://aisevt.shinyapps.io/badoutcomerisk_shinyapp/ (Supplementary Figure S3).

**Table 3:** Performance of the prediction model in the derivation and external validation cohorts.

Metric	Derivation cohort	External validation cohort
Discrimination		
AUC (95% CI)	0.793 (0.754–0.832)	0.712 (0.635–0.789)
Sensitivity	0.768	0.679
Specificity	0.659	0.609
Accuracy	0.720	0.652
Calibration		
Hosmer–Lemeshow χ^2^	3.850	14.145
*P* value	0.870	0.078
Clinical utility		
Decision curve analysis	Positive net benefit across thresholds	Positive net benefit across thresholds
Model comparison		
DeLong test (vs. LASSO model)	*P* = 0.339	*p* = 0.848

Model discrimination was assessed using the area under the receiver operating characteristic curve (AUC). Calibration was evaluated with the Hosmer–Lemeshow goodness-of-fit test. Clinical utility was assessed by decision curve analysis, with positive net benefit indicating superior performance compared with “treat-all” and “treat-none” strategies across clinically relevant threshold probabilities. CI indicates confidence interval.

## Discussion

In this multicenter study of patients undergoing endovascular thrombectomy for acute ischemic stroke, we developed and externally validated a prognostic nomogram integrating atrial fibrillation with rapid ventricular response—a clinical marker of neurocardiac stress—with the Systemic Immune-Inflammation Index. In this exploratory mediation analysis, elevated SII appeared to statistically account for a portion of the observed association between AF-RVR and poor functional outcome, suggesting a potential mediating role of systemic inflammation. These findings are hypothesis-generating and should be interpreted with caution pending replication in future prospective studies.

### Systemic inflammation mediates the prognostic effect of neurocardiac stress

A central finding of this study is that systemic inflammation statistically accounts for a portion of the association between AF-RVR and poor functional outcome. Mediation analysis quantified that the indirect effect through SII was significant, with an absolute risk difference of 4.6 percentage points (ACME 0.046; 95% CI, 0.011–0.084; *p* = 0.018). After accounting for this indirect pathway, the direct effect of AF-RVR was not statistically significant (ADE 0.077; 95% CI, −0.040 to 0.206; *p* = 0.210). These findings suggest that the prognostic signal associated with AF-RVR may be substantially attributable to its relationship with systemic inflammation. However, as with any observational mediation analysis, residual confounding cannot be fully excluded, and these findings should be interpreted as consistent with a mediating role rather than establishing causality. The wide confidence interval for the proportion mediated (37.2, 95% CI, −14.8 to 241.0%) reflects the inherent instability of ratio estimates when the total effect is modest; nonetheless, the significant ACME supports the robustness of the mediation signal.

### AF-RVR as an integrative clinical phenotype of neuro-immune activation

Our findings refine the prognostic interpretation of atrial fibrillation in the post-EVT setting. Patients with AF-RVR were older, had higher baseline NIHSS 23.8±8.9vs.20.3±9.6;P=0.004 , and exhibited greater inflammatory burden: higher prevalence of high SII 64.2%vs.48.0%;P=0.013 and elevated neutrophil-to-lymphocyte ratio 9.3±6.6vs.7.4±8.5;P=0.005 . This clustering suggests that AF-RVR functions as an integrative phenotype signaling concurrent neurocardiac stress and inflammatory activation. Biologically, acute brain injury triggers sympathetic surge—stroke-heart syndrome (6, 9)—manifesting as tachycardia and promoting pro-inflammatory cytokine release (13–15, 21). Admission heart rate was retained as a continuous predictor (aOR 1.040 per bpm; 95% CI, 1.023–1.057), underscoring the prognostic relevance of quantifying neurocardiac tone.

It is important to note that patients with AF-RVR also had a markedly higher proportion of cardioembolic stroke (76.1% vs. 19.9%, *p* < 0.001). Thus, AF-RVR may partly function as a marker of a specific stroke mechanism rather than a purely distinct neurocardiac-inflammatory phenotype. While our multivariable models adjusted for stroke mechanism and other baseline factors, residual confounding by underlying disease severity cannot be fully excluded.

### Systemic inflammation as the dominant prognostic pathway

SII emerged as the strongest independent predictor in the final model (aOR 3.23; 95% CI, 2.13–4.90), exceeding established factors such as age and NIHSS. This aligns with meta-analyses identifying SII as a robust prognostic biomarker in acute ischemic stroke (7, 8, 20). Our study extends these observations by demonstrating SII predicts outcome independent of AF-RVR and heart rate, and by quantifying its mediating role: AF-RVR portends poor prognosis largely because it identifies patients with heightened inflammatory response to brain injury. Post-stroke systemic inflammation exacerbates secondary injury, impairs neuroplasticity, and promotes microvascular dysfunction—contributing to the “no-reflow” phenomenon despite successful recanalization (3, 8, 10, 11).

### Synergistic risk: combined effect of AF-RVR and high SII

Stratified analysis revealed a clinical risk gradient. Relative to patients with low SII and no AF-RVR, adjusted odds of poor outcome were higher with high SII alone (aOR 3.31; 95% CI, 1.20–10.72), further increased with AF-RVR alone (aOR 7.12; 95% CI, 2.34–39.47), and highest with concomitant AF-RVR and high SII (aOR 9.80; 95% CI, 3.46–31.43). Although the multiplicative interaction term was not statistically significant P=0.205 and the model did not show statistically significant improvement in AUC (DeLong test *p* = 0.339), this stepwise escalation identifies a clinically identifiable subgroup with compounded vulnerability. From a translational perspective, this subgroup is easily identifiable using routine admission data and may be optimal for trials of adjunctive immunomodulatory therapies.

### Clinical implications: inflammation as a therapeutic priority

The mediation analysis suggests that the prognostic impact of AF-RVR is substantially statistically accounted for by systemic inflammation, supporting two therapeutic considerations. First, systemic inflammation is a priority target for immunomodulation. SII was the strongest predictor and mediated a substantial proportion of AF-RVR-associated risk. Patients with elevated SII constitute an easily identifiable, high-risk subgroup suited for trials of short-course immunomodulatory strategies, aligning with growing evidence that inflammation is a modifiable determinant of stroke recovery (3, 7, 9). Second, heart rate as a modifiable target. Admission heart rate independently predicted poor outcome (aOR 1.040 per bpm), positioning tachycardia as a bedside indicator of neurocardiac stress and a potential therapeutic lever.

### Strengths and limitations

Strengths include a biologically informed design, rigorous model development with LASSO selection and bootstrap validation, external temporal validation, and mediation analysis providing insight into potential pathways.

### Several limitations warrant consideration

First, the retrospective design introduces potential for unmeasured confounding. Second, AF-RVR was defined using a single admission heart rate measurement. In the hyperacute stroke setting, heart rate can be influenced by pain, agitation, and transient catecholaminergic surges unrelated to sustained arrhythmia burden. Furthermore, we lacked systematic data on the proportion of patients with sustained AF versus rate-controlled AF, or on rate-control therapy use prior to admission. Future studies using continuous cardiac monitoring would enable more refined phenotyping. Third, although we adjusted for multiple clinical covariates, we cannot exclude the possibility that the association between AF-RVR and poor functional outcome is partly mediated through cardioembolic mechanisms rather than neurocardiac stress alone. Future studies with systematic stroke etiology classification and subgroup analyses restricted to AF patients comparing RVR versus non-RVR are warranted to clarify this relationship. Fourth, in the mediation analysis, the proportion mediated was estimated with a wide confidence interval that crossed zero; while the ACME was significant, this ratio estimate should not be interpreted as a precise quantitative summary. Fifth, the interaction analysis may have been underpowered to detect modest synergistic effects. Sixth, this study did not examine sex as a potential effect modifier, despite accumulating evidence that post-stroke inflammatory responses exhibit sexual dimorphism (22–25). Future studies should address this gap. Seventh, although a multicenter design was employed, the participating centers are predominantly located within the same geographic region. This regional concentration may introduce selection bias and limit the generalizability of our findings to populations with different demographic characteristics, stroke management practices, or healthcare infrastructure. Validation in cohorts with broader geographic and ethnic diversity is warrantedprospective validation in broader, more diverse populations is needed to establish generalizability. Finally, we did not assess other clinical outcomes such as mortality or symptomatic intracranial hemorrhage, which would provide additional context for the proposed model.

## Conclusion

This study demonstrates that systemic inflammation statistically accounts for a portion of the prognostic association between neurocardiac stress and poor outcome in patients undergoing EVT. We developed and externally validated a nomogram integrating AF-RVR and SII to predict functional outcome. The co-occurrence of AF-RVR and elevated SII identifies a distinct subgroup at highest risk. Implemented as an online calculator, this tool enables early risk stratification and supports development of biomarker-stratified trials targeting systemic inflammation in high-risk patients with evidence of neuro-immune activation.

## Data Availability

The raw data supporting the conclusions of this article will be made available by the authors, without undue reservation.
