# Supplementary material for: Systemic immune-inflammation index predicts post-thrombectomy outcomes and reveals a mediating role in the association between neurocardiac stress and prognosis: a multicenter study
Source: Front Neurol. 2026 May 29;17:1824909. doi: 10.3389/fneur.2026.1824909 (PMC13260588; doi:10.3389/fneur.2026.1824909)
Supplement: Supplementary file 1 [file Table_1.docx]

Supplementary

**Supplementary Tables**

| **Supplementary Table S1. Baseline Characteristics of the Derivation Cohort, Stratified by 3-Month Functional Outcome** | | | | |
| --- | --- | --- | --- | --- |
| **Variable** | **Total (N = 515)** | **Poor Outcome**  **(mRS 3–6, N = 289)** | **Good Outcome**  **(mRS 0–2, N = 226)** | **P value** |
| **Demographics** |  |  |  |  |
| **Male, n (%)** | 352 (68.4) | 194 (67.1) | 158 (69.9) | 0.500 |
| **Age, years, median (IQR)** | 67.0 (58.5–75.0) | 70.0 (60.0–77.0) | 66.0 (56.0–73.0) | < 0.001 |
| **Vascular Risk Factors, n (%)** |  |  |  |  |
| **Hypertension** | 262 (50.9) | 160 (55.4) | 102 (45.1) | 0.021 |
| **Diabetes** | 100 (19.4) | 67 (23.2) | 33 (14.6) | 0.015 |
| **Atrial fibrillation (any)** | 177 (34.4) | 91 (31.5) | 86 (38.1) | 0.120 |
| **Stroke Characteristics** |  |  |  |  |
| **TOAST subtype, n (%)** |  |  |  | 0.110 |
| **1 (Large-artery atherosclerosis)** | 374 (72.6) | 218 (75.4) | 156 (69.0) |  |
| **2 (Cardioembolism)** | 140 (27.2) | 71 (24.6) | 69 (30.5) |  |
| **4 (Other/undetermined)** | 1 (0.2) | 0 (0.0) | 1 (0.4) |  |
| **Circulation, n (%)** |  |  |  | 0.835 |
| **1 (Anterior)** | 321 (62.3) | 179 (61.9) | 142 (62.8) |  |
| **2 (Posterior)** | 194 (37.7) | 110 (38.1) | 84 (37.2) |  |
| **Admission NIHSS, median (IQR)** | 20.0 (12.0–30.0) | 22.0 (15.0–30.0) | 16.0 (11.0–25.8) | < 0.001 |
| **Procedural Details** |  |  |  |  |
| **Onset-to-puncture, min, median (IQR)** | 350 (250–466) | 380 (280–480) | 310 (230–420) | < 0.001 |
| **Procedure duration, min, median (IQR)** | 95 (70–125) | 100 (80–140) | 90 (60–116) | < 0.001 |
| **Thrombectomy attempts, n (%)** |  |  |  | 0.047 |
| **1** | 342 (66.4) | 180 (62.3) | 162 (71.7) |  |
| **2** | 94 (18.3) | 62 (21.5) | 32 (14.2) |  |
| **3** | 42 (8.2) | 29 (10.0) | 13 (5.8) |  |
| **4–5** | 6 (1.2) | 3 (1.0) | 3 (1.3) |  |
| **Primary technique, n (%)** |  |  |  | 0.116 |
| **1 (Stent retriever)** | 413 (80.2) | 221 (76.5) | 192 (85.0) |  |
| **2 (Contact aspiration)** | 43 (8.3) | 28 (9.7) | 15 (6.6) |  |
| **3 (Combined)** | 55 (10.7) | 37 (12.8) | 18 (8.0) |  |
| **4 (Other)** | 4 (0.8) | 3 (1.0) | 1 (0.4) |  |
| **IVT prior to EVT, n (%)** | 188 (36.5) | 103 (35.6) | 85 (37.6) | 0.645 |
| **Final mTICI 2b–3, n (%)** | 394 (76.5) | 207 (71.6) | 187 (82.7) | 0.002 |
| **Neuro-cardiac & Inflammatory Markers** |  |  |  |  |
| **Admission heart rate, bpm, median (IQR)** | 79 (69–92) | 85 (74–98) | 74 (66–81) | < 0.001 |
| **AF with RVR (HR >100 bpm), n (%)** | 67 (13.0) | 49 (17.0) | 18 (8.0) | 0.003 |
| **High SII (>1012.23), n (%)‡** | 258 (50.1) | 185 (64.0) | 73 (32.3) | < 0.001 |
| **NLR, median (IQR)** | 5.63 (3.25–9.41) | 6.67 (3.47–10.61) | 4.87 (2.90–8.27) | < 0.001 |
| **PLR, median (IQR)** | 155.8 (109.8–226.7) | 158.6 (111.4–236.7) | 145.4 (108.9–212.6) | 0.129 |
| Footnote: Data are presented as n (%) for categorical variables and median (interquartile range, IQR) for continuous variables. P < 0.05 was considered statistically significant. mTICI (modified Thrombolysis in Cerebral Infarction) was treated as an ordinal variable for comparison. SII (Systemic Immune-Inflammation Index) was calculated as (platelet count × neutrophil count) / lymphocyte count. The cut-off value of 1012.23 × 10⁹/L represents the cohort median. Abbreviations: AF, atrial fibrillation; RVR, rapid ventricular response; EVT, endovascular thrombectomy; IVT, intravenous thrombolysis; mRS, modified Rankin Scale; NIHSS, National Institutes of Health Stroke Scale; NLR, neutrophil-to-lymphocyte ratio; PLR, platelet-to-lymphocyte ratio; SII, Systemic Immune-Inflammation Index; TOAST, Trial of Org 10172 in Acute Stroke Treatment. | | | | |

| **Supplementary Table S2. Baseline Characteristics of the External Validation Cohort, Stratified by 3-Month Functional Outcome** | | | | |
| --- | --- | --- | --- | --- |
| **Variable** | **Poor Outcome (mRS 3–6, N = 112)** | **Good Outcome (mRS 0–2, N = 69)** | **Total (N = 181)** | **P value** |
| **Demographics** |  |  |  |  |
| Age, years, mean ± SD | 67.26 ± 10.80 | 64.86 ± 10.30 | 66.34 ± 10.65 | 0.065 |
| Sex, male, n (%) | 73 (65.2) | 41 (59.4) | 114 (63.0) | 0.436 |
| **Stroke Characteristics** |  |  |  | 0.013 |
| Pre-EVT NIHSS score, mean ± SD | 21.02 ± 9.10 | 17.57 ± 9.96 | 19.70 ± 9.56 | 0.013 |
| **Stroke Etiology (TOAST), n (%)** |  |  |  | 0.159 |
| Large-artery atherosclerosis | 82 (73.2) | 49 (71.0) | 131 (72.4) |  |
| Cardioembolism | 17 (15.2) | 16 (23.2) | 33 (18.2) |  |
| Other determined | 0 (0.0) | 1 (1.4) | 1 (0.6) |  |
| Undetermined | 12 (10.7) | 3 (4.3) | 15 (8.3) |  |
| Circulation (Anterior/Posterior), n (%) | 65/47 (58.0/42.0) | 45/24 (65.2/34.8) | 110/71 (60.8/39.2) | 0.336 |
| **Vascular Risk Factors, n (%)** |  |  |  | 0.996 |
| Hypertension | 73 (65.2) | 45 (65.2) | 118 (65.2) | 0.996 |
| Diabetes | 33 (29.5) | 12 (17.4) | 45 (24.9) | 0.068 |
| Atrial fibrillation | 32 (28.6) | 22 (31.9) | 54 (29.8) | 0.636 |
| **Procedural Details** |  |  |  | 0.043 |
| Onset-to-puncture, min, median (IQR) | 415 (320–585) | 350 (240–520) | 380 (290–540) | 0.043 |
| Procedure duration, min, median (IQR) | 120 (89–138) | 95 (70–120) | 101 (79–130) | 0.004 |
| **Neuro-cardiac & Inflammatory Markers** |  |  |  | 0.212 |
| Admission heart rate, bpm, mean ± SD | 77.82 ± 17.67 | 74.14 ± 12.79 | 76.42 ± 16.04 | 0.212 |
| AF with RVR (HR >100 bpm), n (%) | 11 (9.8) | 6 (8.7) | 17 (9.4) | 0.801 |
| High SII (>1012.23), n (%) | 61 (54.5) | 15 (21.7) | 76 (42.0) | < 0.001 |
| NLR, median (IQR) | 5.08 (3.42–8.13) | 4.08 (2.40–6.82) | 4.72 (2.97–7.49) | 0.027 |
| PLR, median (IQR) | 149.5 (111.5–208.0) | 140.7 (110.5–174.3) | 144.7 (110.5–201.3) | 0.141 |
| Footnote: Data are presented as n (%) for categorical variables and as mean ± standard deviation (SD) or median (interquartile range, IQR) for continuous variables, as appropriate. P values were derived from the Chi-square test (or Fisher’s exact test where expected cell counts were <5) for categorical variables and from the independent-samples t-test or Mann-Whitney U test for continuous variables, as appropriate. *P value for the comparison of successful reperfusion (final mTICI 2b–3) is derived from a test comparing the distribution across all mTICI categories (0, 1, 2a, 2b, 3). Abbreviations: EVT, endovascular thrombectomy; NIHSS, National Institutes of Health Stroke Scale; TOAST, Trial of Org 10172 in Acute Stroke Treatment; mTICI, modified Thrombolysis in Cerebral Infarction; AF, atrial fibrillation; RVR, rapid ventricular response; SII, Systemic Immune-inflammation Index; NLR, neutrophil-to-lymphocyte ratio; PLR, platelet-to-lymphocyte ratio; IQR, interquartile range. | | | | |

| **Supplementary Table S3. Characteristics of the Derivation Cohort Stratified by Atrial Fibrillation with Rapid Ventricular Response (AF-RVR) Status** | | | |
| --- | --- | --- | --- |
| **Variable** | **Non-AF-RVR (N = 448)** | **AF-RVR (N = 67)** | **P value** |
| **Demographics** |  |  |  |
| **Age, years, mean ± SD** | 65.0 ± 12.4 | 72.0 ± 9.5 | < 0.001 |
| **Male, n (%)** | 317 (70.8) | 35 (52.2) | 0.002 |
| **Stroke Characteristics** |  |  | < 0.001 |
| **TOAST subtype: Cardioembolism, n (%)** | 89 (19.9) | 51 (76.1) | < 0.001 |
| **Admission NIHSS score, mean ± SD** | 20.3 ± 9.6 | 23.8 ± 8.9 | 0.004 |
| **Neuro-cardiac & Inflammatory Markers** |  |  | < 0.001 |
| **Admission heart rate, bpm, mean ± SD** | 77.1 ± 14.2 | 108.6 ± 6.5 | < 0.001 |
| **High SII (>1012.23), n (%)** | 215 (48.0) | 43 (64.2) | 0.013 |
| **NLR, mean ± SD** | 7.4 ± 8.5 | 9.3 ± 6.6 | 0.005 |
| **Outcome** |  |  | 0.003 |
| **3-month poor outcome (mRS 3–6), n (%)** | 240 (53.6) | 49 (73.1) | 0.003 |
| Footnote: AF-RVR, atrial fibrillation with rapid ventricular response (heart rate >100 bpm); mRS, modified Rankin Scale; NIHSS, National Institutes of Health Stroke Scale; NLR, neutrophil-to-lymphocyte ratio; SD, standard deviation; SII, Systemic Immune-inflammation Index; TOAST, Trial of Org 10172 in Acute Stroke Treatment. | | | |

| **Supplementary Table S4. Univariable Logistic Regression Analysis for Predictors of Poor 3-Month Functional Outcome After Endovascular Thrombectomy** | | | | |
| --- | --- | --- | --- | --- |
| **Variable** | **Category / Unit** | **OR (95% CI)** | **Statistic (z/t/χ²)** | **P value** |
| **Age** | Per year increase | 1.030 (1.015–1.046) | 3.962 | < 0.001 |
| **Thrombectomy attempts** | Per attempt increase | 1.359 (1.074–1.737) | 2.514 | 0.012 |
| **Onset-to-puncture time** | Per minute increase | 1.001 (1.000–1.002) | 2.814 | 0.005 |
| **Procedure duration** | Per minute increase | 1.008 (1.004–1.012) | 3.748 | < 0.001 |
| **Hypertension** | Yes vs. No | 1.508 (1.064–2.142) | 2.300 | 0.021 |
| **Diabetes** | Yes vs. No | 1.765 (1.123–2.820) | 2.425 | 0.015 |
| **Admission heart rate** | Per bpm increase | 1.037 (1.025–1.049) | 6.175 | < 0.001 |
| **AF with RVR** | Yes vs. No | 2.359 (1.356–4.276) | 2.945 | 0.003 |
| **Pre-EVT NIHSS score** | Per point increase | 1.046 (1.027–1.067) | 4.647 | < 0.001 |
| **NLR** | Per unit increase | 1.034 (1.005–1.068) | 2.150 | 0.032 |
| **High SII** | Yes vs. No | 3.728 (2.589–5.409) | 7.008 | < 0.001 |
| Footnote: P < 0.05 indicates statistical significance. Abbreviations: AF, atrial fibrillation; RVR, rapid ventricular response; EVT, endovascular thrombectomy; NIHSS, National Institutes of Health Stroke Scale; NLR, neutrophil-to-lymphocyte ratio; SII, Systemic Immune-inflammation Index. | | | | |

| **Supplementary Table S5. Causal Mediation Analysis of High SII in the Association Between AF‑RVR and Poor 3‑Month Functional Outcome** | | |
| --- | --- | --- |
| Effect | Estimate (95% CI) * | P Value |
| **Total effect** | 0.123 (0.002–0.258) | 0.046 |
| **Indirect effect (ACME)** | 0.046 (0.011–0.084) | 0.018 |
| **Direct effect (ADE)** | 0.077 (–0.040–0.206) | 0.210 |
| **Proportion mediated (%)** | 37.2 (–14.8–241.0) | 0.050 |
| *Estimates represent absolute risk differences (percentage points) on the probability scale, derived from 1000 bootstrap iterations with bias‑corrected 95% confidence intervals. All models were adjusted for age, thrombectomy attempts >1, onset‑to‑puncture time, procedure duration, diabetes, and pre‑EVT NIHSS score. Abbreviations: AF‑RVR, atrial fibrillation with rapid ventricular response; SII, systemic immune‑inflammation index; ACME, average causal mediation effect; ADE, average direct effect; CI, confidence interval. | | |

**Supplementary Figures**

**Supplementary Figure S1. Variable selection using least absolute shrinkage and selection operator (LASSO) regression.**


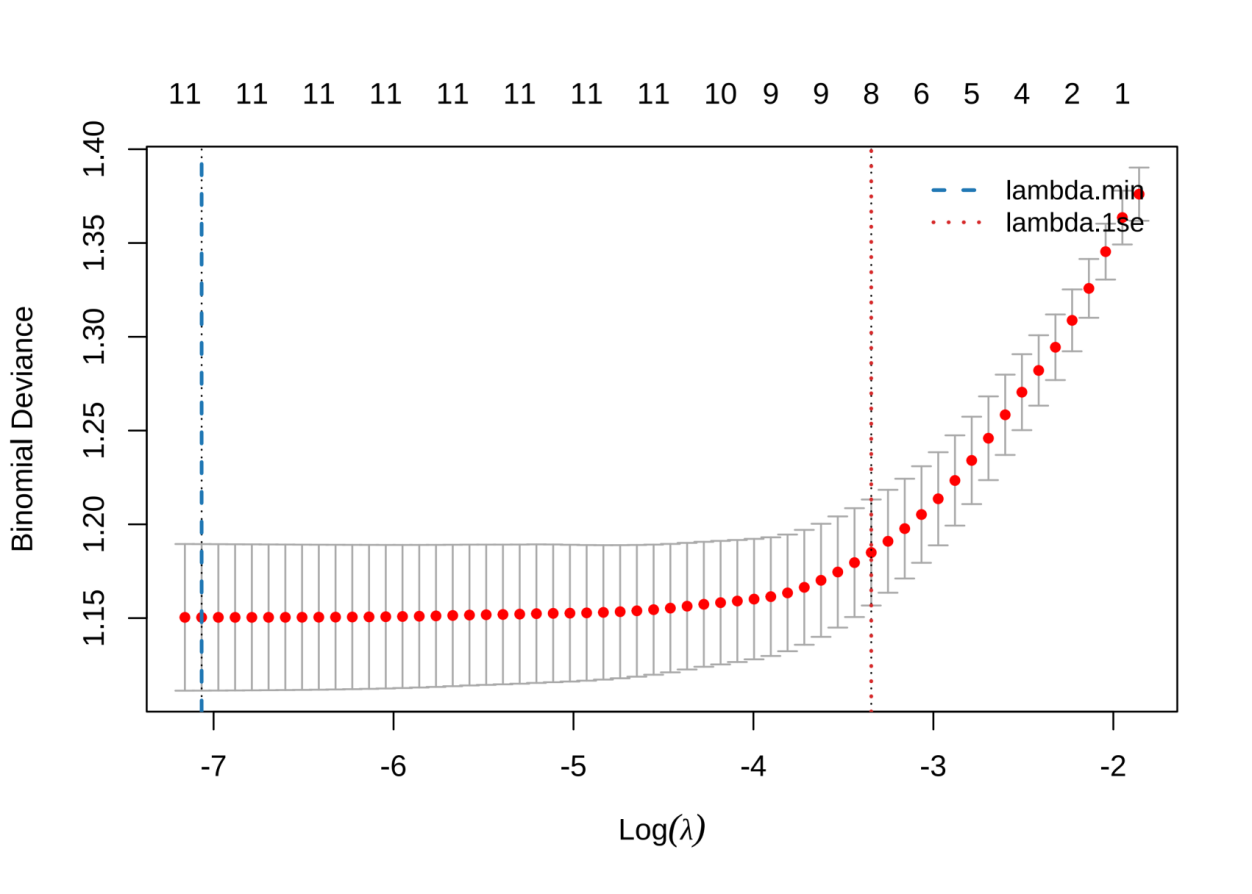


1. **Coefficient paths for the candidate predictors as a function of the regularization parameter (λ).
   (B) Ten-fold cross-validation curve showing the mean binomial deviance. Vertical dashed lines indicate the λ value corresponding to minimum deviance (left) and within one standard error of the minimum (right), with the latter representing the selected model retaining eight predictors.**

**Supplementary Figure S2. Decision curve analysis (DCA) of the nomogram.**

| 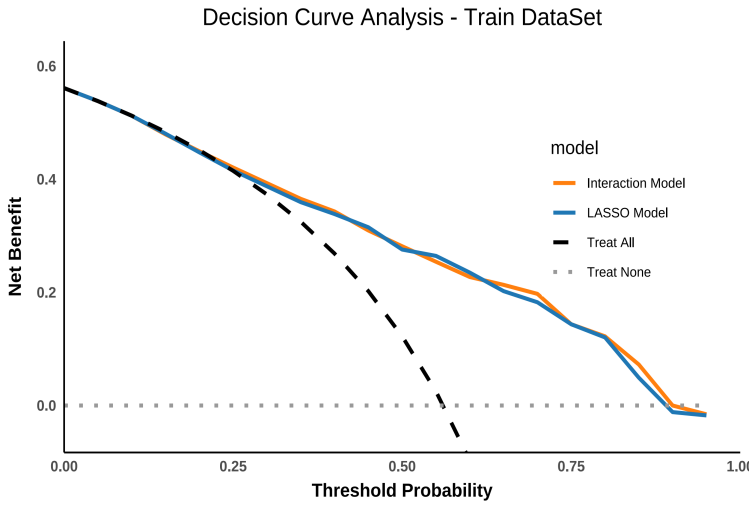  A | 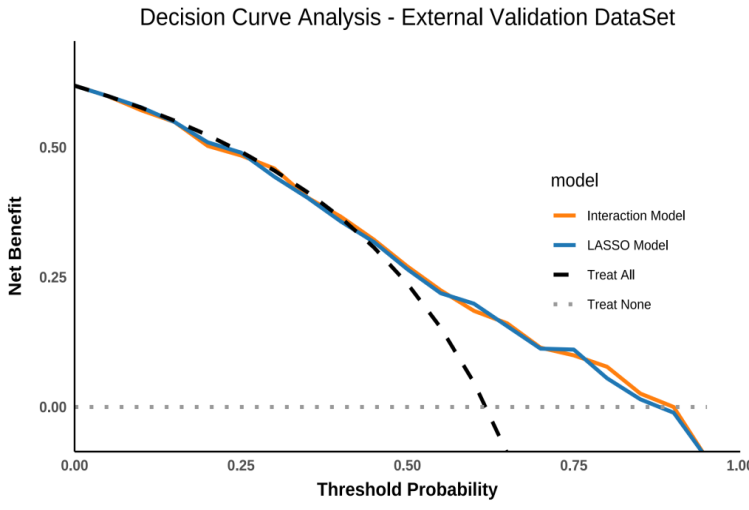  B |
| --- | --- |

(A) Derivation cohort.(B) External validation cohort.

The net benefit of the nomogram (solid lines) is compared with the strategies of treating all patients (black dashed lines) or treating no patients (gray dashed lines) across a range of threshold probabilities.

**Supplementary Figure S3. Interactive online risk calculator for predicting poor 3-month outcome after EVT.**


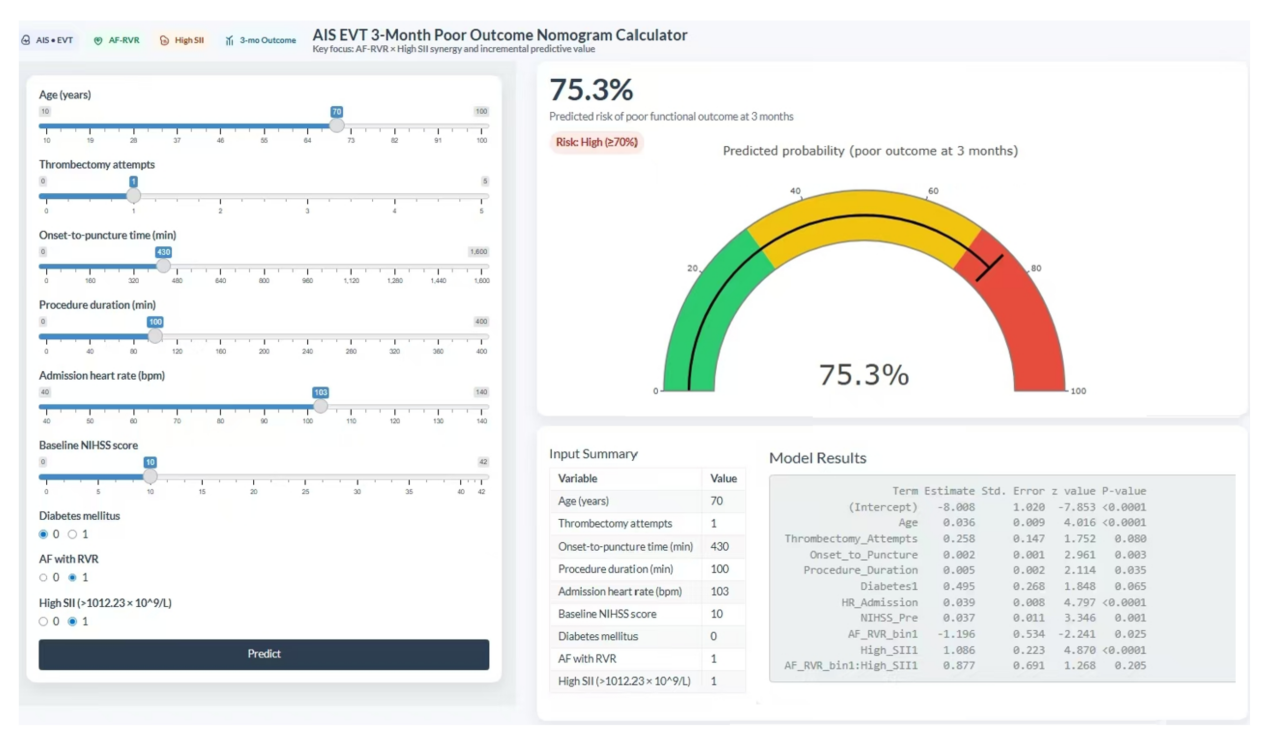

The web-based tool ([**https://aisevt.shinyapps.io/badoutcomerisk_shinyapp/**](https://aisevt.shinyapps.io/badoutcomerisk_shinyapp/#/chat/_blank)) allows users to input predictor variables and obtain a real-time, individualized risk estimate for poor 3-month functional outcome following endovascular thrombectomy.

**Prediction Equation (Logistic Regression):**
logit(P) = -8.008 + (0.036 × Age) + (0.258 × Thrombectomy_Attempts) + (0.002 × Onset_to_Puncture) + (0.005 × Procedure_Duration) + (0.495 × Diabetes) + (0.039 × HR_Admission) + (0.037 × NIHSS_Pre) + (-1.196 × AF_RVR) + (1.086 × High_SII) + (0.877 × AF_RVR × High_SII)
